# Critical assessment of emissions, costs, and time for last-mile goods delivery by drones versus trucks

**DOI:** 10.1038/s41598-023-38922-z

**Published:** 2023-07-21

**Authors:** Aishwarya Raghunatha, Emma Lindkvist, Patrik Thollander, Erika Hansson, Greta Jonsson

**Affiliations:** 1 Department of Building Engineering, Energy Systems and Sustainability Science, University of Gävle, 801 76 Gävle, Sweden; 2 Independent Business Group Sweden AB, 602 21 Norrköping, Sweden; 3 Division of Energy Systems, Department of Management and Engineering, Linköping University, 581 83 Linköping, Sweden

**Keywords:** Environmental impact, Sustainability, Energy efficiency

## Abstract

Electric drones as an autonomous mode of transport are scaling up to transform last-mile goods delivery, raising an urgent need for assessing impacts of drone transport from a systems perspective. In this paper, we conduct systems analyses to assess the environmental, economic, and delivery time impact of large drones for delivery scenarios to pick-up centers between mid-size cities predominantly in rural areas, and deliveries within city limits compared with electric and diesel trucks. Results show that large drones have lower emissions than diesel trucks for deliveries in rural areas and that drones don’t compete with electric trucks, mainly due to the high energy demand required for take-off and landing for each delivery. Furthermore, we show that electric drones are an economically more cost-effective option than road-bound transport modes such as diesel and electric trucks due to the high degree of automation, and also provide the fastest delivery times. Our analysis provides unique insights that drones can address rapid electrification and emergency applications due to low costs, high flexibility, and fast operations. However, for regulators and practitioners to realize it as an emission-friendly option it is necessary to determine the optimal size of drones, particularly for use cases in urban areas, avoid very low landings for deliveries, and have home deliveries instead of pick-up points.

## Introduction

The transportation sector globally gave rise to over 7 Gt CO_2_ in 2020 which is estimated in the IEA (2021) Net Zero Emission scenario to be reduced to 5.5 Gt CO_2_ by 2030 and 0.7 Gt CO_2_ by 2050 ^1^. This vast reduction in global CO_2_ emissions from the transportation sector is estimated even though freight transports are estimated to increase by a factor of 2.5^1^. Currently, 96% of all medium and heavy trucks in the European Union are diesel-driven^2^. Along with this growth in demand for transportation, demand for fast deliveries has increased significantly due to the growth in e-commerce, which brings about an amplified impact on the environment, particularly in the last part of the supply chain where the load reaches the destination^3^. The environmental impact is mainly due to the last-mile delivery trucks being partially loaded, an increase in delivery frequency, and suboptimal delivery logistics^4^. Such low efficiency and poor environmental performance call for optimizing delivery efficiency via development of new freight systems and usage of vehicles with low-emission fuels^3,5^. This in turn calls for exploring disruptive new technology solutions to phase out use of fossil fuels and reach this massive reduction in CO_2_ emissions in transportation. Though both e-mobility and change in transport modes are key means in the built environment to reach this change according to the International Energy Agency’s Net Xero roadmap, the 2030 and 2050 scenarios for transportation remain conventional and land-based^1^.

One novel solution that is deemed to hold such potential is electric drones, or electric vertical take-off and landing vehicles (eVTOLs) as a mode of transport. While some studies claim no clear environmental benefits^6^, other studies show a vast (94%) reduced energy demand for small drones^7^. The concept, entitled Urban Air Mobility (UAM) begun in recent years to address transportation issues in dense cities^8^. It was estimated that the introduction of drones could bring new conditions to transportation systems in and around urban areas, where some of the possible benefits could be traffic reduction, time savings, environmental relief, and improved accessibility^9^. Quickly, the potential for use of such drones in a wide range of transportation use cases in urban, suburban, and rural regions has been recognized under an umbrella term called Advanced Air Mobility (AAM)^10^.

Studies show that environmental benefits of AAM would be greater in rural areas^11^, better than fossil-fueled trucks^12^ and electric trucks^13^, and the estimated effectiveness of drones deliveries to be within shorter distances^14^. Small drones delivering in urban areas are seen to have higher energy consumption due to high customer density and low delivery distances^15^. However, optimally routing drone logistics is suggested to improve energy efficiency and carbon emissions^16^. While drone delivery is shown to be cost efficient for the provider^16,17^, there is conjecture that they may be very expensive and unaffordable for people^18^ and possibly lead to increase in emissions in urban use cases and possibly give more incentive for urban sprawl^19^ in growing mid-sized cities. One main advantage of drone deliveries is stated to be speed and reduction of delivery times^20^. Impacts of drone delivery is context-dependent and vary upon the system in which they are employed^14^ and emissions are greatly dependent on the drones’ energy requirements, distance travelled, and number of deliveries^21^ with energy being a critical constraint in maximizing the benefits of using drones^22^. Despite such uncertainty there is research suggesting the potential benefits of promoting green image of drone deliveries to cater to certain consumer demographics and increase profitability^23,24^. As this field gains increased attention, there is an urgent need for systems analysis of drone impacts for different delivery scenarios for environmental, economic, and delivery time impact in order to make the relationship between the impacts of drone delivery and the context of drone use clear^9,25^.

Therefore, the aim of this paper is to address this gap by evaluating the systems performance of an electric drone for parcel deliveries in mid-sized cities in urban and rural environments. We perform a systems analysis with focus on environmental and economic impacts as well as delivery time. We calculate CO_2_ emissions to assess the global warming potential and conduct life cycle cost (LCC) assessment to uncover the economic impacts. The functional unit (FU) is an important basis for environmental assessment and enables alternative services or goods to be compared and analysed^26^. In this study the FU was chosen as one standard delivery of 250 packages, weighing 2 kg each, divided into three stops, with maximum payload of 500 kg. The calculations are made for drones as well as road-bound delivery vehicles that run on (1) electricity (e-truck), and (2) diesel (diesel truck) in urban and rural scenarios. The system included last-mile deliveries from a logistics center to a pick-up point for both the drone and the alternative road-bound vehicles. Accordingly, four scenarios are evaluated: (1) long route between cities (LBC); (2) short route between cities (SBC); (3) long route within city (LWC); and (4) short route within city (SWC). These estimations provide critical insights regarding the impact drones may have in serving as a disruptive new technology solution to bring down CO_2_ emissions in transportation. Furthermore, sensitivity analysis was conducted particularly for varying energy use and reduced distance in connection with the environmental impact, the number of deliveries in connection with economic impact, and the increase in velocity for delivery time. It should be noted that drones are in an early phase of development, and as for renewable technologies such as photovoltaics that experience rapid advancement until they reach a certain level of maturity^27^ the same can be expected for drones. Thereby we take an ex-ante approach to predict the possible transitions in transport technologies in upcoming decades to determine the factors related to the necessary advancement of technology and the role it will play in society, as urgently needed during early adoption.

## Results

This section presents the findings of the environmental impact assessment for the use phase, economic impact assessment for the life cycle, and delivery time for the chosen electric drone, e-truck, and diesel truck. It was found that overall, in the use phase, the electric drone competes with diesel trucks, but the e-truck is the option with the least environmental impact. In the life cycle of vehicles, the electric drone is the most economically feasible and the fastest alternative. The three assessments and sensitivity analysis of critical parameters are presented separately.

### Environmental impact assessment

**Figure 1:**
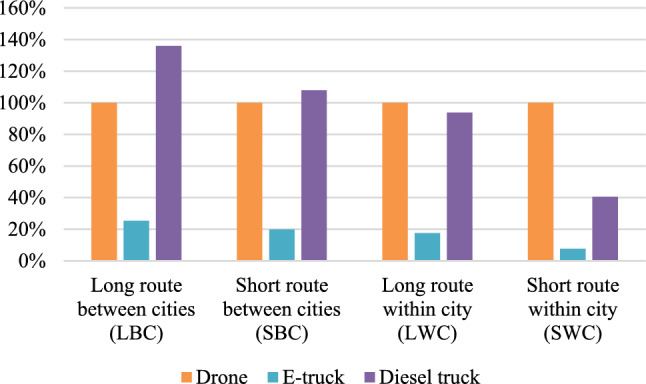
Results from the environmental assessment, where the greenhouse gas emissions per functional unit are presented relative to the greenhouse gas emissions caused by the drone per functional unit.

In Fig. 1, the results of the environmental assessment are presented. We set the result for the drone to 100% for all routes and present the results for the trucks in relation to this. In all cases, the e-truck has significantly lower GHG emissions per FU in all cases. This can be explained by the low energy demand per km compared with the drone and the diesel truck. We find that the environmental performance of the drone is more comparable to the diesel truck where the drone has lower GHG emissions than the diesel truck for LBC by 36% and SBC by 8% but higher GHG emissions for LWC by 6% and SWC by 59%. However, e-truck outperforms environmentally by a minimum of 75% to a maximum of 92%.

The results indicate that the environmental performance of the drone improves when the distance increases. This is mainly due to the reduced energy demand per km for the drone for the longer cases, where the efficient cruise mode is utilized to a higher extent. Since the two trucks follow the same roads in all routes, the only difference between the e-truck and the diesel truck is the fuel consumption and the emission factor for the fuel. Both aspects are larger for the diesel truck compared with the e-truck, which is reflected in the difference in their estimated emissions.

### Economic impact assessment

**Figure 2:**
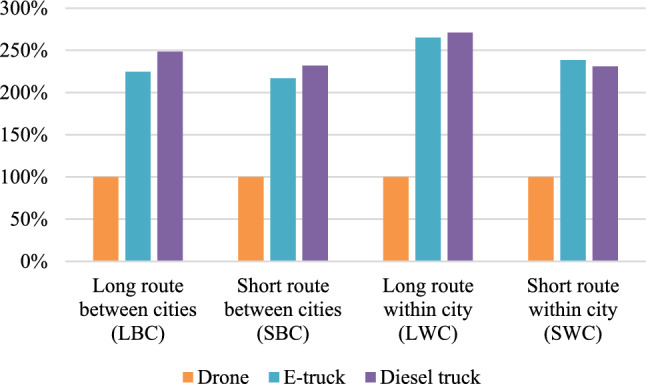
Results from the economic assessment, where the lifecycle costs per functional unit are presented relative to the lifecycle costs of the drone per functional unit.

The results for the economic assessment are presented below in Fig. 2, where costs over the life cycle per FU are presented, and we set the outcome for the drone to 100% and the outcomes for the other vehicles are in relation to the drone. We observe that the drone significantly has the lowest LCC per FU for all routes, while the LCC for the trucks are in the same magnitude. Drones were cheaper than electric trucks by a minimum of 117% and cheaper than diesel trucks by a minimum of 131%.

**Figure 3:**
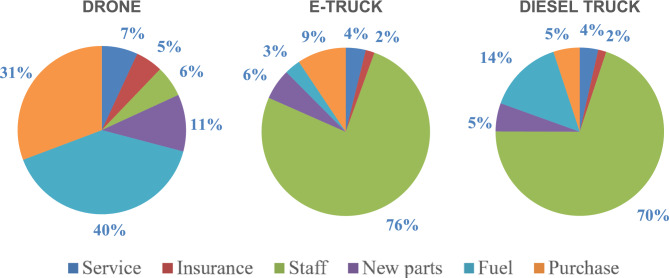
Lifecycle costs split for each vehicle during their lifetime.

The results indicate that drone overall has less than half of the cost per FU in all cases compared with the other vehicles. The largest difference is however seen in the LWC scenario, where the drone has 2.5 times lower LCC per FU. We visualize the reasons for the differences in the LCC for each vehicle by presenting the average distribution of LCC of each vehicle in Fig. 3 below. The largest cost during the lifetime of the drone is from fuel consumption (40%), followed by purchase cost (31%). The largest cost for both trucks is however from staff (76% for the e-truck and 70% for the diesel truck). We therefore conclude that the biggest reason for the lower LCC for the drone is due to cost savings for staff.

Since fuel costs are high for the drone during its life cycle, we find that parameters linked to this are of key importance. The drone is most favorable compared with the other vehicles when the distance saved is longer. This can explain why the LBC scenario is more beneficial for the drone than the SBC scenario, where the reduced distance is 23% compared with 14% in the shorter route. The same reasoning can be used for the routes within cities, where the reduced distance for LWC scenario is 39% versus 30% for SWC scenario, which is an explanation for the lower costs compared with the other vehicles in this route. As stated earlier, the energy usage per km also differs between the routes depending on the distance between the stops, which affects the cost for fuel on the routes. Other variable parameters affecting the results for the economic assessment are factors like cost for service and cost for new parts which vary depending on distance.

### Delivery time assessment

**Figure 4:**
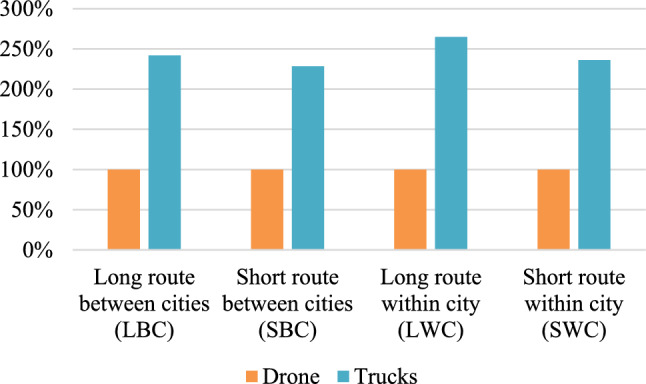
Results from the assessment of delivery time, where the delivery time for trucks per functional unit is presented relative to the delivery time of the drone per functional unit.

The results of the assessment regarding delivery time are presented in Fig. 4, where the results per FU are presented in relation to the drone for all routes. The delivery time for the drone is seen to be evidently less than the delivery time of both trucks in all cases by a minimum of 128%.

Indeed, more time is saved by the drone since the reduced distance is larger for free flying without pre-determined flightpaths. The most time effective route, LWC scenario, is the route in which the most distance is reduced (39%) and the least time effective route, SBC scenario, is the route with the least distance reduced (14%). Furthermore, the reduction in delivery time per FU is affected by the velocity of the vehicles. The speed limits are in general lower in urban areas which leads to a bigger difference between the drone and the trucks regarding speed in these cases. However, we find that the velocity does not seem to have as big an impact as the results for reduced distance.

### Sensitivity analysis

The results from the three assessments have been assessed for varying emission factors and energy usage for all vehicles, the distance taken, number of deliveries made, and velocity assumed for the drone.

#### Sensitivity analysis for emission factor

**Figure 5:**
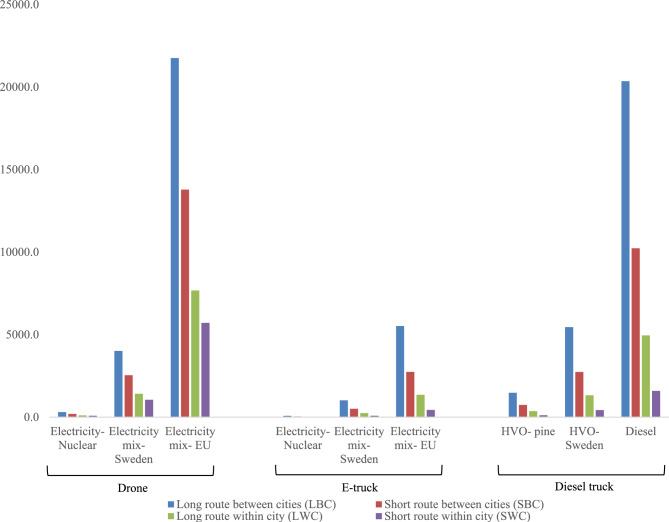
Sensitivity analysis using the low, base case, and high estimation of emissions factors for the fuels used by vehicles under consideration.

The results from the sensitivity analysis for different emission factors for the vehicles for each scenario are presented in Fig. 5 below. We varied the emission factors by assuming different primary fuel from each vehicle. For electric drone and truck primary fuel, the base case emission was calculated assuming primary fuel to be electricity mix from Sweden. Therefore, low estimation was made using electricity from nuclear power and high estimation using European electricity mix. For diesel trucks however lower emission factors were considered by assuming hydrogenated vegetable oil (HVO) from Sweden for medium estimation, and HVO from pine oil for low estimation, since existing combustion engine trucks are expected to transition to biofuel use in the next decade.

The origin of electricity indeed has a big impact on the results. The GHG emissions for the drone increase by approximately 5 times when using the higher estimation compared with the base case and decrease by approximately 12 times when using the low estimation. Compared with the truck fueled by diesel, the drone charged with a Swedish electricity mix is better in all cases. Drone charged with Swedish electricity mix has slightly lower emissions than truck fueled with Swedish HVO for BC scenarios, however the opposite is true for WC scenarios. E-trucks, however, remain less affected since the energy demand per km is low. Although electricity from nuclear and HVO pine seem to have the lowest emissions for the use case of all vehicles, they raise concerns for other sustainability-related issues over their life cycle.

#### Sensitivity analysis for energy use and distance

**Figure 6:**
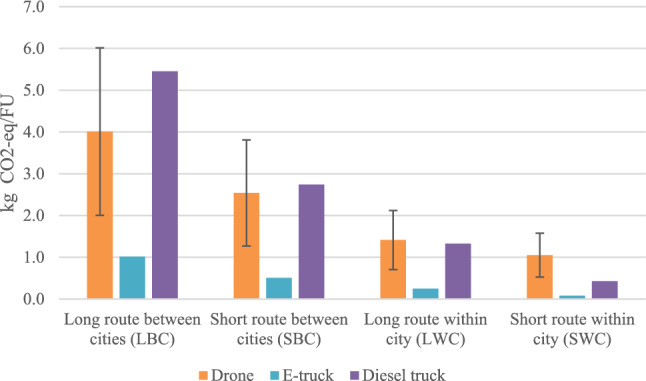
Greenhouse gas emissions from varied energy usage for the drone. The black lines represent ± 50% of the energy usage calculated in the main results.

Variations for the environmental impact are dependent on the energy demand per km of the drone as visualized in absolute values in Fig. 6 below. The black line for every bar of the drone represents the environmental impact if the energy usage per km is either increased or decreased by 50%. When we assume the energy usage of the drone to be 50% higher, the environmental impact of the drone is greater than both trucks in all cases. If the energy usage of the drone instead is decreased by 50%, the drone has lower emissions than the diesel truck for all routes, except the SWC scenario, but still higher than the e-truck for all cases.

**Figure 7:**
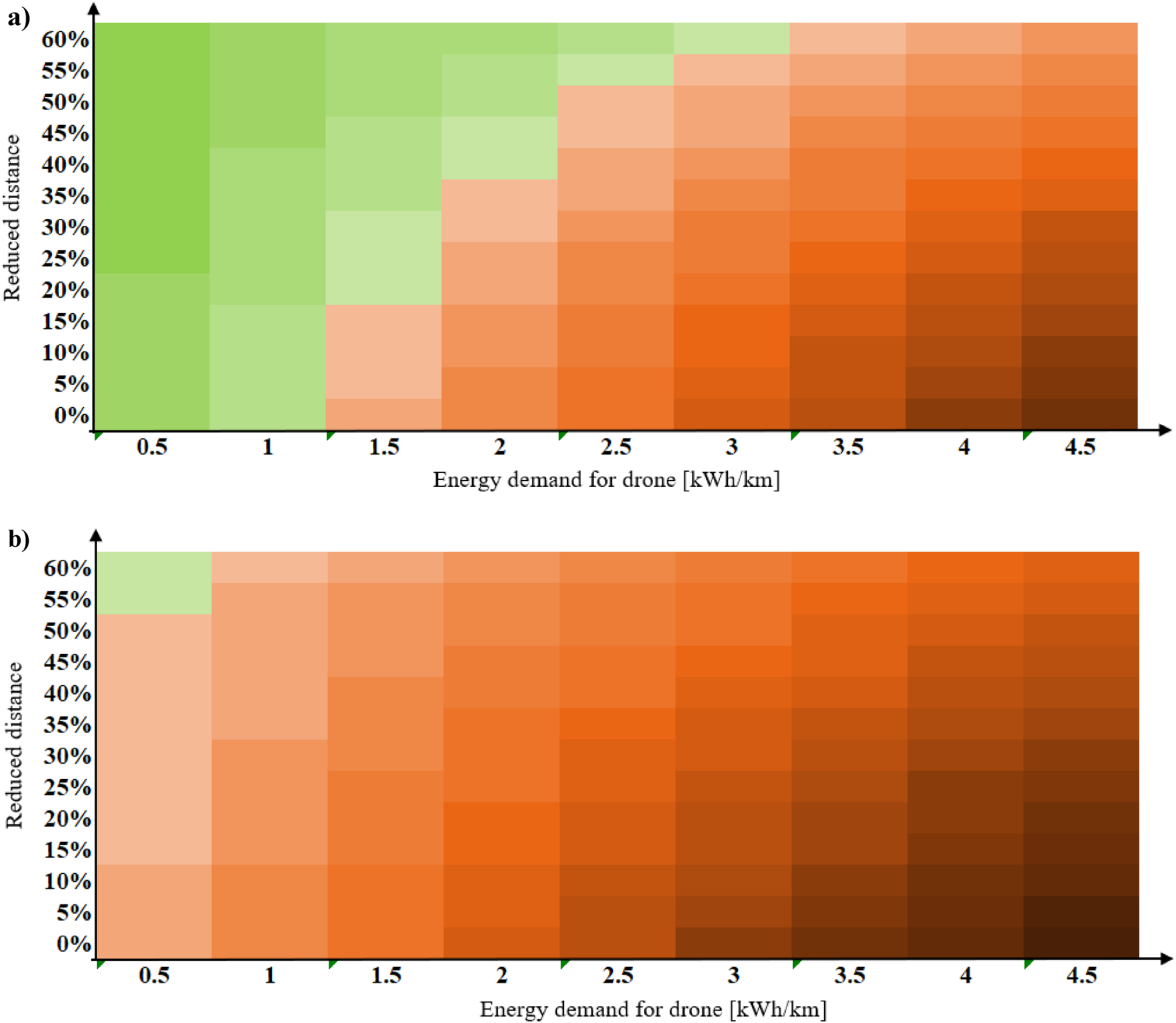
Illustration of the increased (red) and reduced (green) emissions for the drone compared with (**a**) e-truck and (**b**) diesel truck, where the energy demand and distance for the drone is varied, darker colour indicates larger increase/reduction.

Since both the drone and the e-truck use electricity as fuel, the emission factor is assumed to be the same (Swedish electricity mix). Therefore, the only factor that differentiates them regarding environmental impact is the energy usage per km and the distance. The environmental impact of the drone is seen to be lower than the impact from the trucks, the distance reduced either needs to be sufficiently large and/or the energy usage of the drone needs to be sufficiently low. To get a better understanding of the relation between reduced distance and energy usage, these two parameters were combined in a break-even analysis. See Fig. 7a, b where the drone is compared with an e-truck and diesel truck. We varied the energy usage per km between 0.5 and 4.5 kWh/km, and the distance reduction between 0 and 60% to include all the values obtained in the results. A green box indicates that the drone has lower GHG emissions than the alternative vehicle, while a red box indicates an increase.

It is seen that the drone generally causes larger GHG emissions than the e-truck and generally lower GHG emissions than the maximum emission value of diesel truck for a variation of value. The drone starts to perform better than e-truck when energy usage is reduced to and the distance reduced by the following: 3 kWh/km, and the distance reduced by 60%, 2.5 kWh/km and 50%, 2 kWh/km and 40%, 1.5 kWh/km and 15%. Further reduction in energy usage and distance makes drones better perform. We note from this that drones must then have energy usage lower than 1.5 or 1 kWh/km, which would be the energy usage of smaller drones than the one in consideration. Since the energy usage per km for the e-truck is only 0.23 kWh/km, and it is assumed that the energy demand per km for a drone of this size will be 0.5 kWh/km at the lowest, the reduced distance for the drone needs to be at least 55% shorter than for the e-truck for the drone to be the better option. In that case, less than 0.1 kg CO2-eq/FU can be saved. For the worst-case scenario, when it is assumed that the reduced distance is 0% and the energy usage per km is 4.5 kWh/km, the drone accounts for more than 2 kg CO2-eq/FU compared with the e-truck.

When comparing the drone to the diesel truck, the drone accounts for less GHG emissions in all cases compared. Even when the reduced distance is 0%, and the energy usage is 4.5 kWh/km, the drone decreases the GHG emissions by less than 0.1 kg CO2-eq/FU compared with the diesel truck. For the best-case scenario of the drone, when the reduced distance is 60% and the energy usage is 0.5 kWh/km, the drone causes more than 2 kg CO2-eq/FU less than the diesel truck.

#### Sensitivity analysis for varied number of deliveries

**Figure 8:**
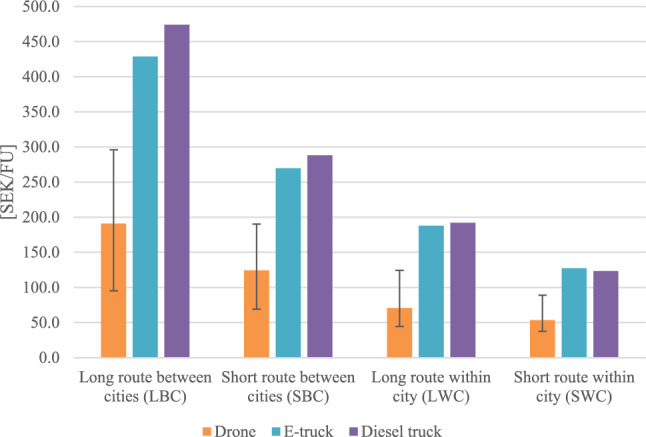
Results for lifecycle costs per functional unit depending on the number of deliveries made by the drone.

In the base case, we assume all vehicles perform delivery missions 8 h a day, where the number of deliveries depends on the delivery time which means that the drone can perform more deliveries per year than the truck, making the solid costs lower per FU. In the sensitivity analysis, we set the lower limit to the same number of deliveries per year as the trucks, assuming the demand of parcel deliveries to be limiting. We set the higher limit of deliveries to 24 h a day instead of 8 h a day, neglecting the time needed for charging. The result from the analysis is presented in Fig. 8 where the lower limit of deliveries is represented by the higher LCC per FU and the increased number of deliveries gives a reduced LCC.

The results of the sensitivity analysis show that when drones deliver all day, costs are reduced by almost 100%. With a decrease in number of deliveries to the same number as trucks, costs increase by close to 100%. Even with this 100% increase in costs, the drone still has lower costs than the trucks in all cases. This indicates that the economic result for the drone can be changed significantly without the cost exceeding the trucks.

#### Sensitivity analysis for varied velocity

**Figure 9:**
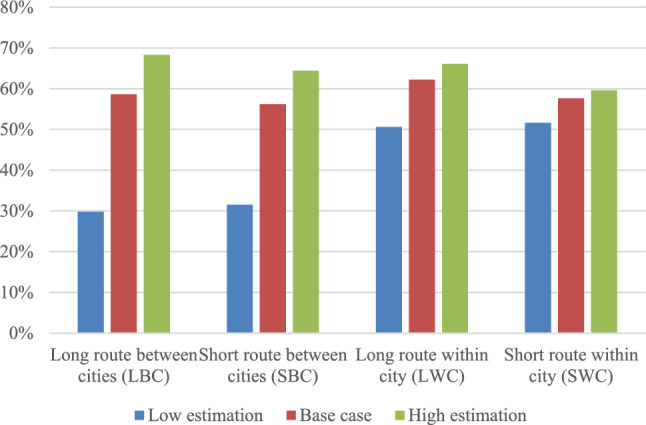
Results for relative time saved per functional unit when using a drone instead of trucks. The low and high estimations represent ± 50% of the velocity in the base case.

We varied the velocity of the drone from 50 to 150% and observed its effect on the delivery time but did not vary the velocity of the trucks since they are bound by speed limits. This relative decreased time of drones compared with trucks is presented in Fig. 9 below. The results are shown for base case, low and high estimation (50% and 150% respectively) for all scenarios. Even when the velocity is lowered by 50% from the base case, the delivery time of the drone still outperforms that of the trucks.

The relative time saved by using drone for low estimation is the lowest for LBC scenario, and highest for SWC. High estimation shows drone to have highest time saved for LBC scenario and lowest for SWC. However, for all scenarios, even the low estimates provide significant time savings. The decreased time per FU is always positive, hence, we observe that delivery with drone is faster than delivery with trucks even if the velocity is reduced by half. This further reinforces that the most important parameter for the decreased delivery time is the shorter distance that the drone uses for deliveries and not the velocity of the drone. It is also natural that the decrease in delivery time is larger in the longer routes, i.e., for BC scenarios, than for the shorter routes, i.e., WC scenarios, because the absolute numbers are generally larger for these routes and additionally reduced distance is longer. It can further be seen that more time per FU is saved when going from the low estimation to base case, than from base case to the high estimation. The BC scenarios are more affected by the change of velocity compared with the shorter WC scenarios, which is a consequence of the longer distance travelled in these cases.

## Discussion

The primary objective of this study was to fill a gap in scientific systems analysis of drone deployment for delivery by undertaking a comparative analysis of drones with electric trucks and diesel trucks for a case study conducted in mid-sized cities. The results of this paper show the emissions, life-cycle costs, and delivery time delimited to the use phase of the vehicles compared for two mid-size cities. Typically for transportation and city planners, the paper shows the importance of understanding various factors that come into play to determine the most suitable options for future transportation design. Implicit decision-making for these actors occurs within the frames of environmental mobility goals, citizens’ needs, and business interests. Results show that it is crucial to understand that the impacts of drones are highly use-case dependent, primarily based on factors explored in this study.

The key findings of this study show that out of the three vehicles, e-trucks consistently emerge as the option with significantly lowest emissions. In contrast, the drone has the highest emissions for the scenarios within city limits and the diesel-fueled truck has the highest emissions in the scenario between two cities. This study indicates that drones compete directly with diesel trucks when it comes to environmental impact. This has to do with the energy efficiency of the vehicles. Notably, the study is carried out without including the infrastructural requirements for the different transport modes, i.e., construction and maintenance of roads needed for the trucks and vertiports for drones.

Irrespective of the scenarios, drones surprisingly turn out to be the most economically efficient option. The major cause for this is due to the lack of need for staff to operate the drones. Lastly, drones are also the fastest option in all cases due to their flexibility as an air mode of transport. The findings from this paper in part contradict the results from Rodrigues et al. (2022)^7^, which shows drones to be able to reduce emissions by up to 94%. Such difference in results could be dependent on the choice of the drones, putting its application in a real-systems context in varying topographies, and getting a fair comparison based on the future technological capacities of the vehicles in comparison. This contradiction also shows that the results of this paper can be generalizable only for drones carrying up to 500 kg in and around mid-sized cities.

In this study, the nature of delivery points in the delivery scenarios between cities, where drones can be considered instead of diesel-fueled trucks, is within rural areas, as supported by previous studies^11^. Rural areas, which struggle with infrastructure issues and lower loads as well as the lower frequency of package deliveries, can be an effective place to consider the use of drones. However, the use of drones is indicated in the industry to begin within urban areas so that a bigger market can be reached, and the business segment can obtain a higher chance of developing the technology, infrastructure, and services. The issue with such a development scenario, in light of drones having the highest emissions in delivery scenarios within urban environments while being the most cost-effective and fastest option, is that this can create increased competition in business, thus increasing interest of business and end users, creating lock-ins that are difficult to change over time, particularly within complex urban infrastructures. This can lead to increasing and even worsening emissions due to the convenience of enhancing the use of the delivery system.

Drones could create a possibility to achieve more optimized load factors than trucks since they are not dependent on the ability to carry a pilot. The drone could therefore potentially be improved by adjusting the size of the drone according to the size and weight of the parcel, giving a higher load factor, which is not as applicable for trucks. In that case, a smaller drone with less energy demand could be utilized when delivery size does not require more, and a larger one applied when needed. Furthermore, trucks and electric motors are innovations that have been built for a very long time, leading to improved efficiency. However, this is not the case for drones which is an emerging technology, wherefore results from this paper should be seen in this perspective that as drone operations may become more efficient attributable to steep learning curves in its production, results may very well fall in the favor of drones, e.g. as has been the case for PV^27^. Until then it is practical to limit the size of drone based on their application, as suggested by Stolaroff et al., (2018)^14^.

Results show that the emissions from drones reduce with smaller distances, and the use of drones is not yet valid in the same way as conventional logistics systems, i.e., deliveries from the logistics center to pick-up points. Instead, final delivery to customers directly from pick-up points to end users would be a rather valid use for drones. Therefore, the logistics system can be multimodal, drones can complement the use of trucks, and can be flexible and localized to improve efficiency.

Drones are mostly acclaimed for their ability to provide delivery services to end users’ doorsteps. Although this aspect is delimited by this study, the findings of the study nonetheless show that the most energy-using operations are during take-off and landing, while cruise and hover are energy-optimized. This significantly implies that for deliveries at multiple stops using drones, the drones may be used to make rooftop deliveries and not doorstep deliveries, thus reducing take-offs and landings and improving their energy efficiency during the deliveries.

There is a possibility that if the technology readiness levels increase with payload increase, the energy efficiency may not necessarily improve due to higher operational energy requirement^28^. This implies that technology developers must have energy efficiency as the target parameter and not very high payloads. From the findings, it is also evident that from an emission standpoint, tactical drones are comparable with diesel trucks in the evaluated scenarios and not with e-trucks since e-trucks have the highest energy efficiency. This further indicates that while designing logistics models, one might not have to consider drones as a competition to trucks, but rather as complements in specific use cases that are highly time-critical, and/or energy efficient in case of unavailability of e-trucks.

Another context to be considered is the actual transition of delivery trucks to electric. A major share of delivery trucks currently use diesel as the primary fuel^2^. Transition pathways for e-trucks today face challenges such as heavy weights of the trucks, reduced payloads, and reduced distances per charge. Through such operational and human-based challenges, the findings from this paper imply that drones may hold the potential to accelerate the transition from fossil-driven vehicles in intra-city last-mile delivery logistics. The adoption of electric drones can be higher due to cost savings and fast delivery times. This could further enable possibilities for accelerating renewable energy production and charging infrastructure.

The significantly high cost-efficiency for 24-h services and low delivery times implies that drones’ deliveries can be particularly beneficial for cases that involve medical services and deliveries to hospitals and rehabilitation centers. This would help significantly cut costs for healthcare.

The deliveries in the study are limited to deliveries from warehouses to pick-up points, excluding the first mile of the delivery mission, the last delivery to the customer, and the parcels reaching the logistics center from several warehouses. A strong recommendation for future studies is to develop scenarios for the last part of the supply chain, from pick-up point to customer, where the supply chain varies depending on the customers (e.g., distance travelled, vehicle type, and allocation for purpose of the trip).

The values and assumptions considered in this study are based on highly reliable data e.g., peer reviewed publications, vehicle manufacturers’ data, in-depth interviews, and inputs from relevant actors in the field. In-depth explanation and sources for data used for calculations are presented in the “Methods” section and Supplementary material. Due to the scope, this study is delimited to consider combined truck and drone delivery scenarios, which has been previously modeled in transportation research using small drones carried by trucks^29–34^. Given the substantial variation in impacts and reliance on specific scenarios, integrating them with trucks is a possible concept for more reliable and better energy efficient transportation. However, it is important to note that the feasibility of this combined truck-drone approach becomes questionable when considering larger drones with payloads comparable to trucks as done in this study due to size and efficiency. The results of this paper are therefore limited to holding significance while weighing the choice between a drone and a truck of similar payloads for the last-mile delivery. In addition, pricing for drone transportation could be significantly impacted by congestion prices for lower altitude airspace management systems^17,35^. However, the consideration for such pricing when drones are yet to be deployed poses huge uncertainty and depends majorly on the demand for drones which this study is delimited to do. Therefore, we strongly recommend future research to consider multimodal scenarios and drone congestion pricing.

Finally, during the study, it was identified that there is a lack of regulation regarding the velocity of drone flights. This lack of regulatory standards for what the velocity should be during take-off, landing, and cruise needs to be addressed in future studies, to determine the right velocity for safety within flight paths, built infrastructure, nature, impact on package items, and such. This study provides significant implications particularly for transportation and city planners, with respect to impacts that need to be considered while contemplating implementation of drone transportation systems. Indications are provided regarding the consequences of future scenarios, along with insights into considerations regarding what type of applications can be beneficial while designing such logistics. For technology developers, this study emphasizes the importance of focusing on energy efficiency primarily instead of load capacity for improving the impacts of use of drones for delivery. Additionally for regulators a critical policy gap regarding the lack of regulations for the drone during take-off and landing as well as cruising of the drone is identified.

## Methods

**Figure 10:**
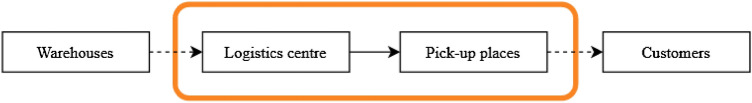
The different parts of a delivery chain. The orange border shows the parts included in this study.

In this paper, a comparison between electric drone, e-truck, and diesel truck was conducted through environmental impact assessment, life cycle cost (LCC) assessment, and delivery time assessments. The functional unit was chosen as one standard delivery of 250 packages, weighing 2 kg each, divided into three stops, with maximum payload of 500 kg. Parcel deliveries using these vehicles from a logistics center to several pick-up points (often located at grocery stores in Sweden) was studied, see Fig. 10. An ex-ante approach was used by predicting the technological advancements for the years 2027–2032, when the usage of drones is more likely to be in a commercial stage, and assuming parameters and scenarios accordingly. The system boundaries were defined individually according to the appropriate parameters for each assessment. The ex-ante approach of this study creates a level of uncertainty for the results, especially for the drone that has not reached a commercial stage in Sweden today. Sensitivity analyses were therefore performed for the results of the assessments to evaluate the robustness of the estimated parameters, and to further analyse the impact of different parameters.

### Scenario description

The established functional unit gives a delivery of approximately 83 packages per pick-up point. To make a representative comparison of the drone and the trucks in different topographical contexts, four scenarios have been formulated in mid-sized Swedish cities Norrköping and Linköping, as follows:

- Long route between cities (LBC)
- Short route between cities (SBC)
- Long route within city (LWC)
- Short route within city (SWC)

Intercity deliveries or route between cities entails deliveries to pick-up points in rural areas between Linköping and Norrköping, where the starting point is Linköping’s logistics center in the north of Linköping and the end point is the logistics center located in the north of Norrköping. Intracity deliveries or routes within cities refer to deliveries in urban and suburban areas in Norrköping and the end point is the same as the starting point.

#### Routes between cities—BC scenarios

**Figure 11:**
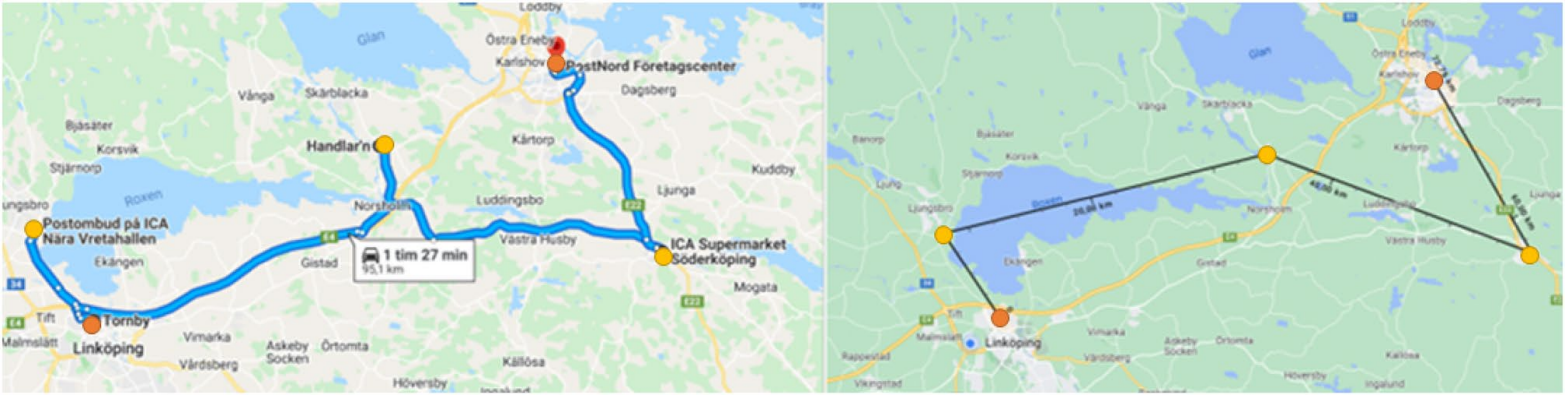
The longer truck route between cities (left) with a total distance of 95.1 km and the drone path (right) with a total distance of 72.8 km (Retrieved from Google Maps^36^, https://goo.gl/maps/ngLZEXyrxQwdQxpk7). The starting and end points are indicated by orange circles and the three stops are indicated by yellow circles.

**Figure 12:**
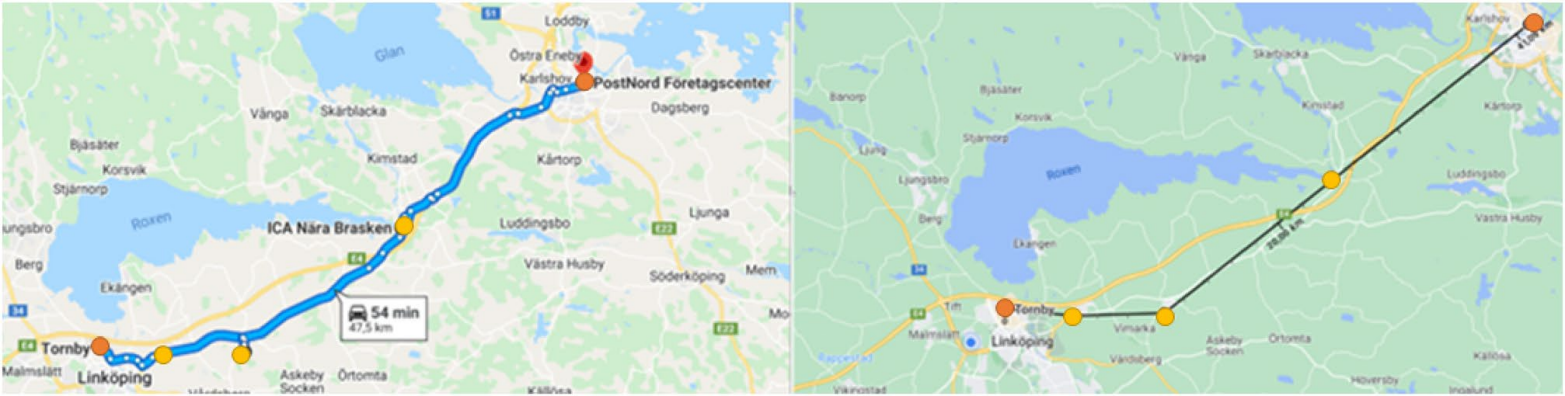
The shorter truck route between cities (left) with a total distance of 47.5 km and the drone path (right) with a total distance of 41.0 km (Retrieved from Google Maps^36^, https://goo.gl/maps/JrSxpXLfeJhwE3XaA). The starting and end points are indicated by orange circles and the three stops are indicated by yellow circles.

In intercity delivery cases packages are delivered from Linköping to Norrköping with stops in smaller villages in between the cities. Two possible routes of intercity deliveries are investigated, where one was longer and included villages further away from the highway connecting the two cities and the other was shorter with stops closer to the highway. Even though the longer route gives a detour for the truck and therefore could be seen as unlikely for real-life delivery-missions, it was seen as interesting variations to include different contexts in the study. The longer route has stops in Ljungsbro, Kimstad and Söderköping before arriving in Norrköping, which gives a total distance of 95.1 km for the road-bound vehicles and 72.8 for the drone. The shorter route has stops in Tallboda, Linghem and Norsholm which gives a total distance of 47.5 km for the trucks and 41.0 km for the drone. The two routes LBC and SBC are shown in Figs. 11 and 12 respectively.

#### Routes within cities—WC scenarios

**Figure 13:**
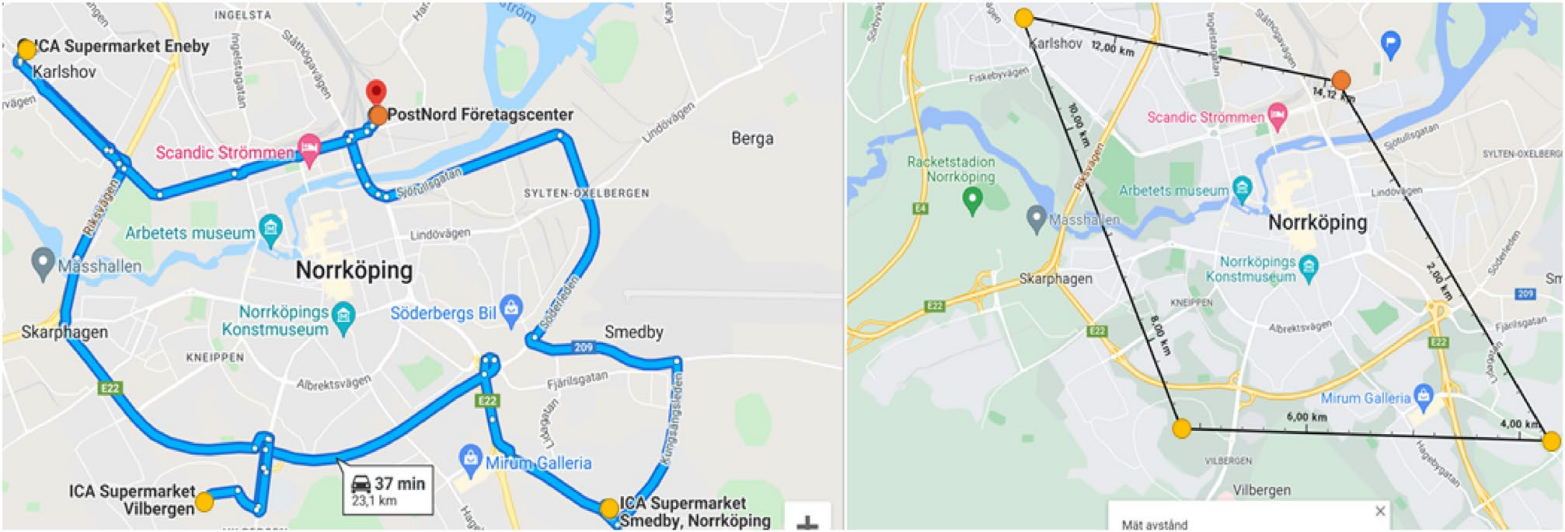
The longer truck route within the city (left) with a total distance of 23.1 km and the drone path (right) with a total distance of 14.1 km (Retrieved from Google Maps^36^, https://goo.gl/maps/XHLHCTVaGgDmxsgM9). The starting and end point are indicated by an orange circle and the three stops are indicated by yellow circles.

**Figure 14:**
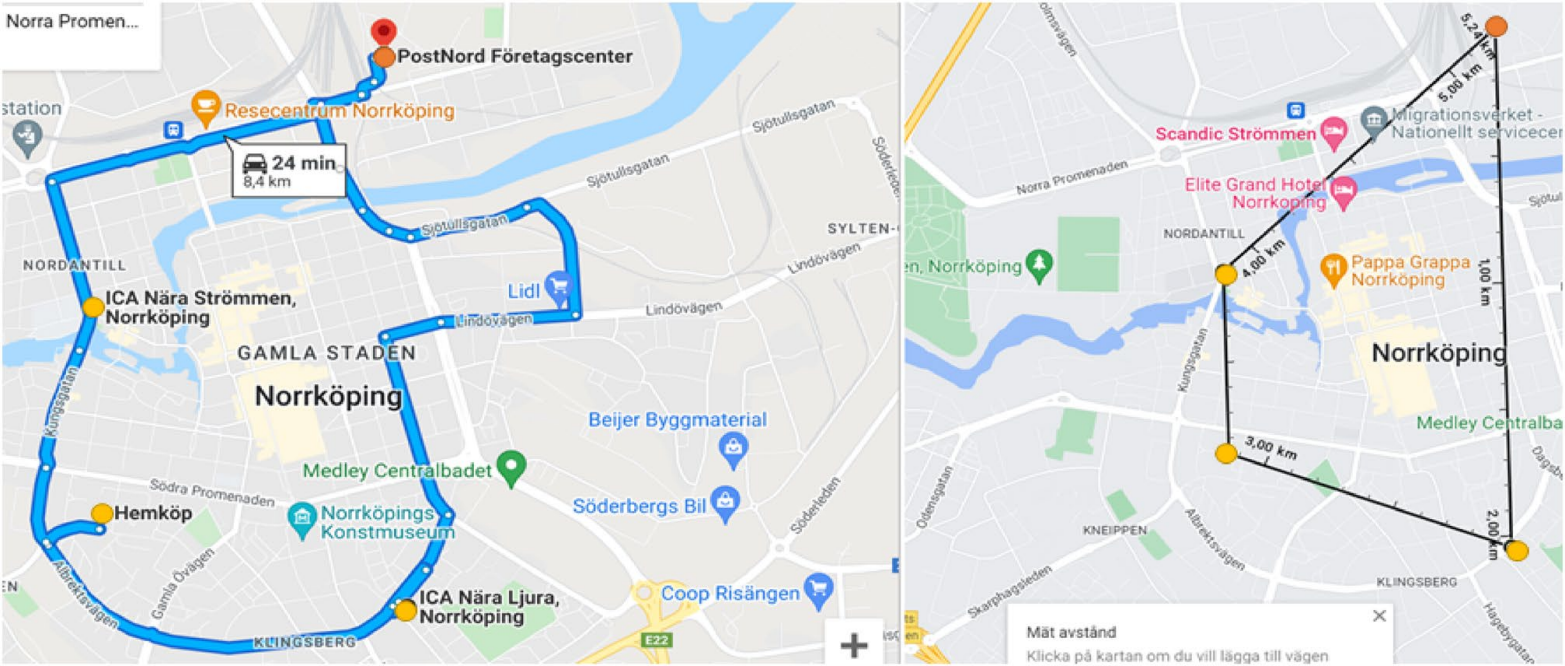
The shorter truck route within the city (left) with a total distance of 8.4 km and the drone path (right) with a total distance of 5.2 km (Retrieved from Google Maps^36^, https://goo.gl/maps/jACU616etC9YkbeY7). The starting and end point are indicated by an orange circle and the three stops are indicated by yellow circles.

Two different routes for the second case, deliveries within Norrköping, were computed where one included transport to the more suburban areas, and one was focused on the city center. Both routes depart and arrive at the logistics center in Norrköping. The different routes for the two cases and different vehicles are shown in Figs. 13 and 14 below. The longer route (LWC gives a total distance of 23.1 km for the trucks and 14.1 km for the drone, and the SWC gives a total distance of 7.4 km for the trucks and 5.2 km for the drone). The stops were combined into routes suitable for the urban and suburban scenario based on where the pick-up places in Norrköping are located today.

### Electric drone under consideration

The parameters for the drone chosen in this study are mainly based on the conceptual electrical “side-by-side” (see Fig. 12) designed by Johnson et al. (2018)^37^. This eVTOL is based on a series of studies performed by NASA to analyse the drone’s requirements for future AAM system and the type of use cases expected^37–39^. The electrical side-by-side is a duo-copter with a range of 137 km (and a reserve of 20-min cruise mode). It is a tactical drone with the ability to carry a payload of 544.3 kg. The drone is designed to carry people, but since it has the desired capacity for this study it is assumed to be representative for deliveries of parcels without a pilot, complete specifications for which are provided in supplementary material. eVTOL manufacturers estimate the price of the drone to be between 0.6 and 1.5 MSEK while the lifetime of the drone is estimated to be 10,000 flight hours. Based on 2024 yearly flight hours (8 h a day for 253 days) this would equal a lifetime of five years with no residual value at the end. See Table 1 for more technical information about the electric drone.

**Table 1:** Specification of vehicles used in the study.

Condition	Drone	E-truck	Diesel truck
Fuel	Electricity	Electricity	Diesel
Maximum capacity	544.3 kg	846 kg	975 kg
Installed power	428 hp	130 hp	136 hp
Range	137 km	–	114 km
Lifetime	5 years	5 years	5 years
Price	1 MSEK	0.355 MSEK	0.605 MSEK
Residual value	0 MSEK	0.177 MSEK	0.301 MSEK

### Delivery trucks under consideration

Most delivery trucks today run on diesel. However, the ex-ante approach of this study considers the competitive vehicles for technologically advanced drones in the period 2027–2032 for a fair comparison. While road vehicles are largely expected to be electric by this period, diesel trucks are included as well, since currently 96.3% of all medium and heavy trucks in the European Union are diesel-driven^2^. Thus, the two vehicles for comparison with drones are e-truck and diesel truck, with payloads of 975 kg and 846 kg, prices of 0.605 MSEK and 0.355 MSEK, and residual values of 0.301 MSEK and 0.177 MSEK respectively as shown in Table 1. The lifetimes of the trucks were assumed to be 5 years, to facilitate the calculations. Complete specifications of the trucks are provided in supplementary material.

### Energy and emissions calculations

**Figure 15:**
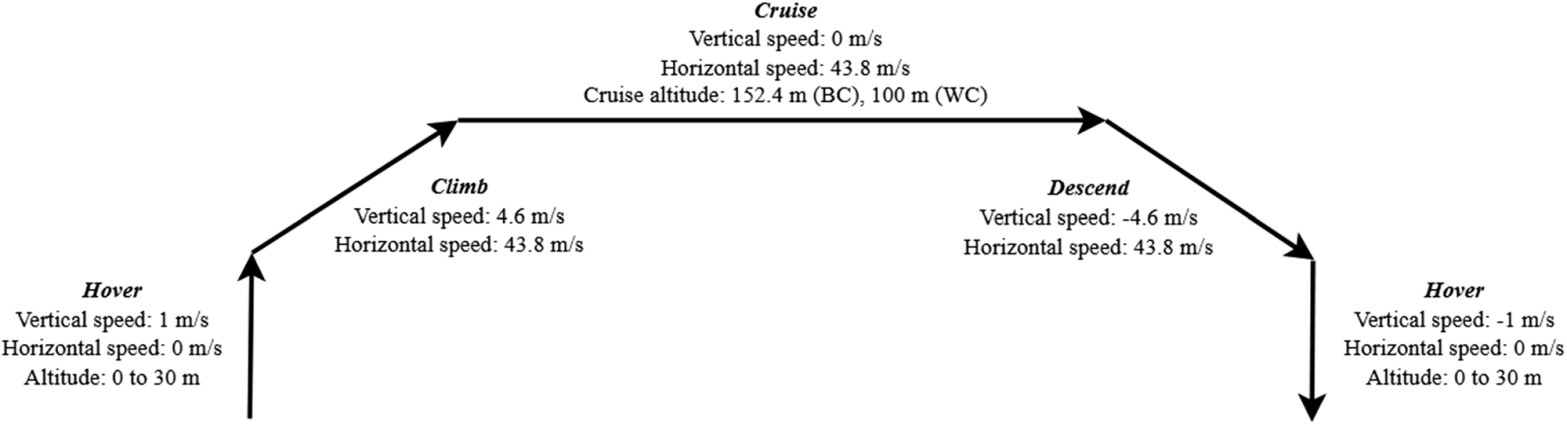
Drone speed vertically and horizontally, and flight altitude during different stages of flight.

In Fig. 15, an overview of operations for the mission of drone flight is presented. There are five stages of drone flight operations: hover, climb, cruise, descend and hover^40^. The flight operations are performed one time for each stop plus one extra to reach the destination. The flight between cities is assumed to be performed at an altitude of 500 feet (152.4 meters). Flights within cities are assumed to be performed at an altitude of 100 meters since the shorter distance between the stops limits the time of climb. The vertical and horizontal speeds in the different stages are assumed from Silva et al. (2018)^39^ where the cruise speed is chosen as the best range speed.

The different stages of the flight require different amounts of energy where hovering and climbing are the most demanding stages. Given that the studied scenarios have different length and duration in the different stages, the energy requirements per km for the drone will differ between the scenarios. Therefore, the total energy needed for drone during one delivery will be the sum of the energy required for each flight operation^40,41^.

Following Kasliwal et al. (2019)^41^, power required for the different stages is calculated using Eqs. (1, 2, 3, 4) which are modelled upon parameters such as sea level air density ($\rho$), disk load of the drone ($\delta$), hover system efficiency (${\eta }_{h}$), climb system efficiency (${\eta }_{cl}$), cruise system efficiency (${\eta }_{cl}$), potential energy conversion from altitude into distance ($L/D$) for cruise, climb and descent, rate of climb ($ROC$), rate of descent ($ROD$, which is − $ROC$), speeds in different phases ($V$), mass of the drone $m$, and gravitational acceleration ($g$=9.82 m/s^2^)^41^. These parameters for the drone in consideration in this paper are provided in supplementary material.

1 $${P}_{hover}= \frac{mg}{{\eta }_{h}}\sqrt{\frac{\delta }{2\rho }}$$

2 $$P_{cruise} = \frac{mg}{{\left( {L/D} \right)}}\frac{V}{{\eta_{cr} }}$$

3 $$P_{climb} = \frac{mg}{{\eta_{cl} }}\left( {ROC + \frac{{V_{climb} }}{{\left( {L/D} \right)_{climb} }}} \right)$$

4 $$P_{descend} = \frac{mg}{{\eta_{cl} }}\left( {ROD + \frac{{V_{descend} }}{{\left( {L/D} \right)_{descend} }}} \right)$$

The energy requirements for drones thus calculated are presented in Table 2. Out of the four scenarios the required energy per km for LBC scenario, which is the longest of the four routes, is the lowest and the required energy per km for SWC scenario, which is the shortest route, is the highest.

**Table 2:** Vehicles’ energy requirements for each scenario.

Scenario	Drone (kWh/km)	E-truck (kWh/km)	Diesel truck (kWh/km)
LBC	1.17	0.79	0.23
SBC	1.32	0.79	0.23
LWC	2.13	0.79	0.23
SWC	4.31	0.79	0.23

The energy requirements per km for the trucks are collected from the manufacturers’ websites and are based on the Worldwide Harmonised Light Vehicles Test Procedure. The value is then adjusted based on the changed load factor during the route. The energy requirement is increased by 5.6 Wh/km per 100 kg additional weight according to the assumption made by Ellingsen et al. (2016)^42^. The load factor has low impact on the required energy for the trucks as shown in Table 2.

The objective of the environmental assessment was to evaluate the impact for global warming potential (GWP) of the scenarios. Each package is assumed to weigh 2 kg, since 75–90% of large companies’ last-mile deliveries weigh below 2.3 kg^13^. The study took a tank-to-wheel approach, assessing the potential environmental impact from the vehicles’ use phase. The system boundary for fuel and electricity was however wider, applying cradle-to-grave, including the environmental impact from production to combustion. To calculate the environmental impact from the different cases and vehicles, emission factors for electricity and diesel were found through the Swedish Environmental Institute^43^, see Table 3. The emission factor for electricity is a complex parameter since it is dynamic depending on the momentary production of electricity and on geographic location. In this study Swedish electricity mix including consideration of import and export was chosen. For gaining the environmental impact from the vehicles’ use phase, the emission factors were multiplied by the vehicle’s fuel consumption and the distance of the route.

**Table 3:** Emission factors for fuels used.

Fuel	Emission factor (g CO2eq/kWh)
Electricity	47
Diesel	273

### Life-cycle costs calculation

In the economic assessment, the aim was to get an overview of the costs for each of the vehicles. Therefore, a life cycle cost analysis (LCCA) was performed using the equation for net present value (NPV). The NPV represents the present value of all costs and benefits linked to an investment where the future costs are discounted to present value^44^. The net present value benefits from the vehicles’ operations are however excluded due to the study being conducted for a future scenario. Therefore, the most economically beneficial alternative in the study is represented by the alternative with the lowest costs during their life cycle. The equation for calculating the NPV^44,45^ is presented below.

5 $$NPV=\sum Net\,present\,value\,benefits-\sum\,Net\,present\,costs-\sum\,Investment\,costs$$

**Figure 16:**
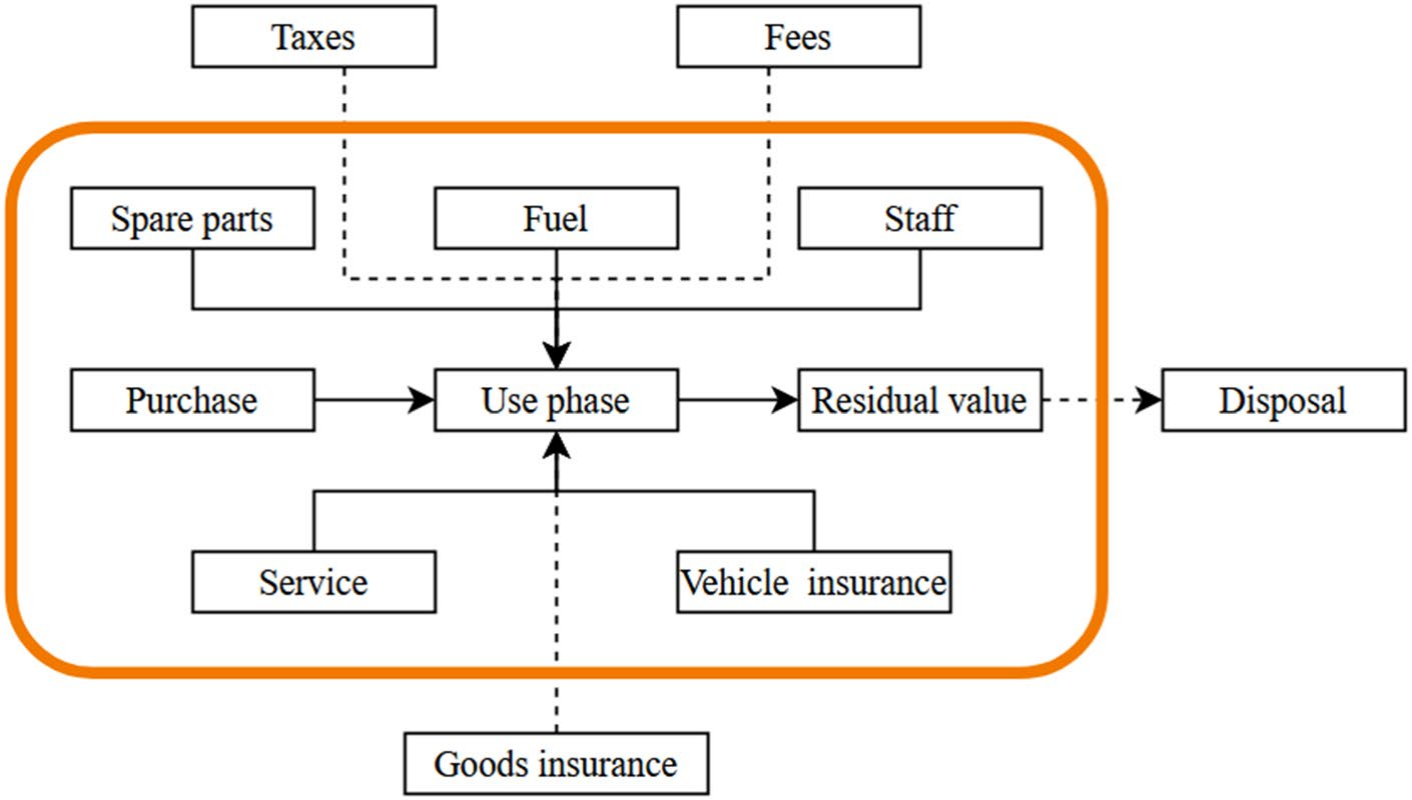
The system boundaries for life cycle cost assessment of the vehicles, where the orange border shows the parts included in this study.

To discount future values, the discount rate was set to 12% based on a recommended required rate of return of 10% and 2% inflation^43^. Furthermore, most costs are assumed to be nominally unchanged and are based on today’s values (excluding value-added tax). The values assumed with today’s values are then recalculated with the rate of inflation to each year of the study period, except fuel costs which are assumed to increase above inflation, and battery cost which is expected to decrease from current value. Costs included in the study are initial costs, service and maintenance cost, operating costs, disposal costs, and vehicle insurance costs. These costs differ for each alternative vehicle and hence are deemed important; other costs that are uncertain in terms of predictability such as taxes, fees, and insurance of goods are excluded. Thus, the system boundaries for LCC conducted is shown in Fig. 16. The summary of all cost parameters used is shown in Table 4. For more details regarding procurement of parameters see supplementary material.

**Table 4:** Summary of all the cost parameters used in the economic assessment.

Parameter	Value
Discount rate	12%^43^
Service cost	0.33 SEK/km^43^
Cost of new tires	0.51 SEK/km^43^
Cost of new batteries	755 SEK/kWh^46^
Electricity price (2027–2032)	1.1–1.2 SEK/kWh^43^
Diesel price (2027–2032)	15.6–17.3 SEK/liter^43,47^
Cost of truck driver	40 929 SEK/month^48,49^
Cost of drone operator	97 537 SEK/month^48,49^
Insurance cost of trucks	11 000 SEK/year
Insurance cost of drone	34 000 SEK/year

### Delivery time calculations

The delivery time was defined as the time from departure at one logistic center, via deliveries to three pick-up points, to arrival at the other logistic center for each route and vehicle. The assessment includes both time for driving and delivery. We assume that both electric and diesel trucks drive at the same speed limits and on the same route in all cases. Hence, the delivery times for drones are identical. To estimate the time of driving for the trucks, the fastest route was used during non-congested times on Google Maps. The drone is assumed to fly the shortest possible path between the nodes at a constant speed (see scenario description). Drop-off with a truck is assumed to take an average time of 3 min^13^. It is furthermore assumed that the drone will land on the ground when delivering, requiring extra time for take-off and landing. The time required for the drone to lift and land is set to 1 min. The procedure consists of 30 s hovering down from an altitude of 30 meters and then 30 s hovering up to an altitude of 30 meters. The extra minute is then added to the delivery time for the drone, making the total delivery time 4 min for the aerial vehicle.

### Sensitivity analysis calculations

Parameters that were tested in sensitivity analysis were:

- Emission factor—for all vehicles
- Energy usage—for the drone
- Reduced distance by the drone
- Numbers of deliveries—for the drone
- Velocity—for the drone

Emission factor, energy usage and reduced distance when using a drone instead of road-bound vehicles, are important categories for the study in general, and especially for the environmental assessment since these parameters affect the consequences of the use phase. The emission factors are a critical parameter for all vehicles since it is dependent on the future market scenarios and production of fuel. To see how the results were affected by different scenarios, the emission factor for electricity was changed to both higher and lower values, and the emission factor for diesel was changed to two different lower values. The emission factor for electricity was adjusted to a higher value corresponding to the electricity mix in the EU in 2019, and a lower value corresponding to electricity from nuclear power. The diesel truck can also be fueled with HVO without conversion and therefore, the emissions corresponding to HVO mix in Sweden 2020 are used as a medium estimate. The lower case for the diesel truck is HVO made from pine oil. The values used for the different fuels can be found in Table 5.

**Table 5:** Summary of all the cost parameters used in the economic assessment.

Scenario	Estimation	Type of fuel	Emission factor (g CO2eq/kWh)
Alternatives for diesel	Base case	MK1-diesel	273^50^
	Medium	HVO, Swedish mix 2020	73^50^
	Low	HVO from pine oil	19.8^51^
Alternative for electricity	High	Electricity mix, Europe 2019	255^42^
	Base case	Electricity mix, Sweden 2020	47^50^
	Low	Electricity from nuclear power	3.6^52^

Energy usage per km and reduced distance when using an electric drone are vital parameters for the environmental assessment. To see the robustness of the results and to find break-even points, sensitivity analyses were performed where different energy and reduced distance were varied to see when the electric drone is beneficial. Break-even analyses were also performed for an e-truck and a diesel truck, as well as a truck fueled with HVO, to get a broader picture of the variations on the environmental impact of the electric drone when comparing to different vehicles.

To test how much the number of deliveries affects the results from the economic assessment, the number of deliveries made by the drone were set to a higher and a lower value. The lower limit is set to the same number of deliveries per year as the trucks, assuming the demand for parcel deliveries to be limiting. The higher limit of deliveries is set to 24 h a day instead of 8 h a day, which was assumed to be possible since the drone is unmanned. The number of deliveries made during a lifetime impacts the results for economic assessment where the impact is total cost per standard delivery based on the functional unit for the lifetime of the vehicles.

For delivery time, the velocity of the drone during cruising and climbing is an uncertain parameter due to current lack of legal standards. Sensitivity analysis for higher (increased by 50%) and lower (decreased by 50%) velocities of the drone is conducted to understand the impact on delivery time. It is assumed that the drone flies at constant speed in all cases while the speed of the truck remains constant since it is dependent upon established speed limits.

### Supplementary Information

Supplementary Information.

## Data Availability

All data generated or analysed during this study are included in this published article [and its supplementary information files].
