# Author Correction: Critical assessment of emissions, costs, and time for last-mile goods delivery by drones versus truck

**DOI:** 10.1038/s41598-023-41725-x

**Published:** 2023-09-02

**Authors:** Aishwarya Raghunatha, Emma Lindkvist, Patrik Thollander, Erika Hansson, Greta Jonsson

**Affiliations:** 1 Department of Building Engineering, Energy Systems and Sustainability Science, University of Gävle, 801 76 Gävle, Sweden; 2 Independent Business Group Sweden AB, 602 21 Norrköping, Sweden; 3 Division of Energy Systems, Department of Management and Engineering, Linköping University, 581 83 Linköping, Sweden

Correction to: *Scientific Reports* https://doi.org/10.1038/s41598-023-38922-z, published online 21 July 2023

The original version of this Article contained an error in Figure 15, where the values for vertical and horizontal speed in the cruise phase and the vertical speed in the hover phase for landing were incorrect.

The original Figure 15 and its accompanying legend appear below.

**Figure 15:**
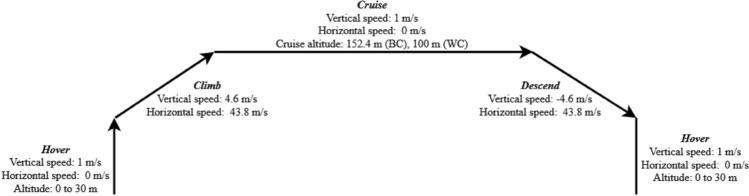
Drone speed vertically and horizontally, and flight altitude during different stages of flight.

The original Article has been corrected.

