# Supplementary material for: Critical assessment of emissions, costs, and time for last-mile goods delivery by drones versus trucks
Source: Sci Rep. 2023 Jul 21;13:11814. doi: 10.1038/s41598-023-38922-z (PMC10362012; doi:10.1038/s41598-023-38922-z)
Supplement: Supplementary file 1 — Supplementary Information. [file 41598_2023_38922_MOESM1_ESM.docx]

**Supplementary material**

**Electric drone specifications; NASA conceptual Electrical “Side by side” [1]**

| Fuel | Electricity |
| --- | --- |
| Mass empty body | 1672.4 kg |
| Total installed power | 428 |
| Maximum capacity | 544.3 kg |
| Battery capacity | 846 |
| Range | 137 km |
| Hover efficiency | 0.63 |
| Cruise efficiency | 0.765 |
| Measure of potential energy conversion from altitude into distance | 7.2 |
| Velocity in cruise mode | 43.8 m/s |
| Disk load | 167.6 N/m^2^ |
| Rate of climb | 4.57 m/s |
| Rate of descend | -4.57 m/s |

**Table S1.** Specifications and energy parameters for drone considered in the study [1]

**E-truck specifications; Volkswagen E-crafter [2]**

| Fuel | Electricity |
| --- | --- |
| Energy usage | 21.5 kWh/100km |
| Maximum load value | 10.7 m^3^ |
| Maximum load mass | 975 kg |
| Total installed power | 130 hp |
| Battery capacity | 35.8 kWh |
| Range | 114 km |
| Price | 604,700 SEK |

**Table S2.** Specifications for e-truck considered in the study [2]

**Diesel truck specifications, Transit 2.0 l EcoBlue L3 Trend [3]**

| Fuel | Diesel, HVO |
| --- | --- |
| Energy usage | 8.2 (1/100km mixed driving) |
| Maximum load value | 15.1 m^3^ |
| Maximum load mass | 846 kg |
| Total installed power | 136 hp |
| Maximum effect | 96 kW |
| Price | 355,068 SEK |

**Table S3.** Specifications for diesel truck considered in the study [3]

**Procurement of cost parameters**

The first phase of the LCCA is the purchase of the vehicle where we collected the information about retail prices from the manufacturers of the vehicles. Since the studied drone is not yet constructed, the exact retail price cannot be established. To be able to include this, we based the cost on the future estimation of the price made by a drone manufacturer.

The next phase is the use phase where most of the costs appear. Both service and maintenance costs, and operating cost are linked to the use phase of the vehicle. Our assumption for the estimated cost for service is 0.33 SEK/km for all vehicles [4]. Note that the vehicles travel different distances and therefore have different service costs. The estimation is made for trucks but since no information regarding service costs for drone could be found, the cost was set to the same level as the truck. The service cost for drone was then adjusted in the sensitivity analysis to determine the uncertainty of the assumption. Linked to the service costs are the maintenance cost where the cost for spare parts is included. The spare parts included in the calculation are tires and batteries since these are deemed the most important factors. The cost of new tires is set to 0.51 SEK/km [4]. Furthermore, electrical vehicles used in the study is affected by the life time of the batteries used. A Li-ion battery is said to typically be recharged 1000-3000 times before replacement [5], therefore 2000 cycles with 80 % depth of discharge (DOD) is used in this study. The market for batteries is, however, developing fast and the price for new batteries are therefore an uncertain parameter. It is predicted that the price of Li-ion batteries will be between 61-100 U.S. dollars/kWh in 2030 [6], where an average value of this is used for calculations. The average value is approximately 80 U.S. dollars/kWh which is estimated to be 755 SEK/kWh.

The next part of the calculations of the costs in the use phase is the operating costs including cost for fuel and staff. The cost of fuels, both electricity and HVO were determined for the future as follows. The electricity price for companies, excluding the value-adding tax, where set to 0.90 SEK/kWh in 2017 and is estimated to be 1.4 SEK/kWh in the year 2040 [4]. We calculated the electricity price for each year between 2037-32 based on these values and the assumption that the rate of increase will be stable from 2017 to 2040. The price of diesel and HVO is estimated to increase by 1.5 % per year until 2040. With this rate of increase and an average price of diesel and HVO for companies (excluding the value-adding tax) set to 13.74 SEK/liter for the year 2019 [7] and the yearly price of diesel corresponding to the studied period (2027-2032) could be calculated. The cost of fuels is further tested in the sensitivity analyses.

The cost for staff, linked to the road-bound vehicles are limited to the driver of the trucks. The salary for a driver is assumed to be 29 500 SEK/month based on the average salary for a Swedish truck driver [8]. The cost of staff for the drone includes the salary of the operator controlling the flights. We assumed that the operator could control 30 drones at the same time based on the information received from the drone manufacturers. The salary is corresponding to the salary of a pilot since a drone operator will be able to monitor several drones and be a person with a regular license of a pilot [9]. The average salary for a pilot in Sweden today is 70 300 SEK/month [8]. The salaries were then adjusted to represent the cost of staff for the company, adding on fees and insurances, making the cost of the truck driver 40 929 SEK/month and the operator 97 537 SEK/month [10]. The value of the salaries represents today’s levels but is adjusted to higher values according to inflation in the calculations.

The last cost included in the use phase is the cost of insurance for the vehicles. We estimated the insurance cost for trucks (including full insurance and free deductible) to be 11,000 SEK/year based on our e-mail correspondence with an insurance intermediary from the County Insurance body in Sweden regarding insurance cost of trucks. The drone manufacturers we contacted suggest the insurance cost for the drone regarding injuries on the vehicles itself is 1000-2000 SEK/year and mandatory insurance cost for potential injuries on other vehicles is estimated to 2-4.5 % of the vehicle purchase cost. In this study we use the average value, resulting in a cost of 34 000 SEK/year, a rough estimation based on a general value for an aircraft flying mainly over land (not water), carrying 500 kg. However, it could be assumed that the insurance cost for drone operations is higher than for a ground-based vehicle since it is associated with a higher risk in less controlled environments of weather conditions and wind. Insurance cost for the cargo is as stated excluded from the study for all vehicles.

The last phase of the LCCA is the disposal phase where this study is limited to the residual value of the trucks. The decrease in value for the truck is estimated to 13 % of the retail price per year [4]. Since the technology of drones is novel there is not yet a second-hand market for drones and their residual value is assumed to be zero. The disposal costs for the vehicles are not included in the assessment since the uncertainties linked to the novel technology of drones is deemed too big.

**References**

1. Silva, C., et al., *VTOL Urban Air Mobility Concept Vehicles for Technology Development.*, in *AIAA Aviation and Aeronautics Forum (Aviation 2018)*. 2018: Atlanta, GA.

2. Volkswagen. *Volkswagen e-Crafter. Electric van and light truck.* 2022; Available from: <https://www.volkswagen-transportbilar.se/sv/innovationer-och-teknik/elbilar/modeller.html>.

3. Ford. *Prislista Ford Transit Custom Skåp, Dubbelhytt i skåp, Kombi och Buss.* 2022; Available from: <https://fordbusiness.se/wp-content/uploads/2020/02/Transit-Custom-produktfakta.pdf>.

4. Administration, S.T., *Analysis methodology and socio-economic costings for the transport sector: ASEK 7.0.* 2020.

5. Analysis, T., *Development of the road vehicle fleet by 2030*. 2020.

6. BNEF. *Battery Pack Prices Fall As Market Ramps Up With Market Average At $156/kWh In 2019*. 2019.

7. Preem, *List prices business cards*. 2020.

8. Sweden, S. *Salary statistics - How much do you earn...?* 2020 27-04-22]; Available from: <https://www.scb.se/hitta-statistik/sverige-i-siffror/lonesok/>.

9. Sudbury, A.W. and E.B. Hutchinson, *A Cost Analysis Of Amazon Prime Air (Drone Delivery).* Journal for Economic Educators, 2016. **16**(1): p. 12.

10. Verksamt. *Calculate the cost of an employee*. 2020 [cited 27-04-2022; Available from: <https://www.verksamt.se/alla-e-tjanster/rakna-ut/rakna-ut-vad-en-anstalld-kostar?p_auth=hrzMSxBR&p_p_id=tvv_webcalc_WAR_tvv_webcalc&p_p_lifecycle=1&p_p_state>=.
